# Sequential Human Assembly and Disassembly Motions in Human-Robot Coexisting Environments

**DOI:** 10.1038/s41597-025-06042-0

**Published:** 2025-11-11

**Authors:** Zhihao Liu, Tianyu Wang, Zhenrui Ji, Wenjun Xu, Lihui Wang, Xi Vincent Wang

**Affiliations:** 1 Department of Production Engineering, KTH Royal Institute of Technology, Brinellvägen 68, Stockholm, 114 28 Sweden; 2 UM-SJTU Joint Institute, Shanghai Jiao Tong University, Dongchuan Road 800, Shanghai, 200240 China; 3 School of Information Engineering, Wuhan University of Technology, Luoshi Road 122, Wuhan, 430070 China; 4 Hubei Key Laboratory of Broadband Wireless Communication and Sensor Networks (Wuhan University of Technology), Luoshi Road 122, Wuhan, 430070 China

**Keywords:** Mechanical engineering, Mechanical engineering

## Abstract

As human-robot systems and autonomous robots become increasingly prevalent, the need for task-oriented datasets to study human behaviors in shared spaces has grown significantly. We present a novel dataset focusing on sequential human assembly and disassembly motions in human-robot coexisting environments. It contains over 10,000 samples recorded from multi-view camera setups, each comprising synchronized RGB videos and 2D and 3D human skeletons. Data were collected from 33 participants with diverse physical characteristics and behavior preferences. This dataset highlights practical challenges such as partial occlusions, similar repetitive motions, and varying human behaviors, which are often overlooked in existing datasets and research. Technical validation using benchmarking with state-of-the-art deep learning models reveals significant potential in using this dataset for practical applications. To support diverse research applications, this dataset provides raw and processed data with detailed annotations, including precise timestamps, procedure annotations, and Python codes for reproducibility. It aims to advance research in human motion prediction, task-oriented robotic sequential decision-making, motion and task planning of autonomous robots, and human-robot collaborative policies.

## Background & Summary

A human-robot system (HRS) is a novel paradigm that comprises humans and robots working together as a unified workforce^1^. This paradigm is reflected in key research areas such as human-robot interaction (HRI) and human-robot collaboration (HRC)^2^. An HRS typically consists of fundamental modules, including perception, cognition, decision-making, and control. Among these modules, perception plays a central role. Moreover, the ability of robots to perceive task-oriented sequential manual procedures performed by humans distinguishes perception in HRS from conventional robotic perception.

Typical modalities for capturing human behaviors include RGB images, RGB-D images, videos, point clouds, and human skeletons. These modalities are often employed to track and recognize human activities. By leveraging the real-time states of human operators, task and motion planning can be implemented to enhance the collaborative capabilities of robots, thereby enabling efficient HRS, especially task-oriented ones. However, human motions in HRS, especially HRC, are predominantly task-related, particularly in collaborative assembly and disassembly.

Related datasets exist in both HRI and HRC. In HRI, MHRI^3^ is a multi-modal dataset containing human motion pointing and showing objects to a robot. While THOR^4^ is a motion trajectory dataset for human-robot navigation. Similar datasets in HRI also show up in other robotic-related fields such as hugging interaction^5^, personality and engagement^6^, games^7^, and assistive collaboration^8^. In HRC, the analysis of human motion is often industrial task-oriented, encompassing aspects such as body language and the task sequences involved in manipulation tasks. For instance, InHARD^9^ utilizes webcams and wearable motion capture devices in an HRC scenario where humans and robots operate side by side. HRI30^10^ collects data from an industrial environment in which a human collaborates with two robots, capturing human motions such as “pick up drill” and “move forward while drilling” from a wide camera view. Assembly101^11^ is a large-scale, multi-view dataset designed for sequential manual assembly tasks, primarily developed for augmented reality applications without the inclusion of physical robots, thereby focusing predominantly on forearm and hand movements above a table. ATTACH^12^ emphasizes two-hand assembly actions performed by humans, involving 42 participants in a cabinet assembly task, with data collected using three cameras. HA-ViD^13^ highlights the knowledge in assembly from 30 participants, with video data collected from three cameras.

These datasets released in recent years have significantly contributed to HRS research. However, they still present limitations in supporting HRS for industrial tasks. Firstly, common human motion datasets, such as Kinetics^14^ and NTU RGB+D^15^, do not account for task-oriented human behaviors, especially task sequences. They typically focus on simple daily life activities, such as crossing arms or raising hands. As for HRS-related datasets, InHARD relies on wearable devices, which limits its usage because of higher hardware requirements. HRI30 focuses more on humans walking between robots while holding tools, which is less task-oriented. The advantages of Assembly101 include its large scale and multiple camera views; however, it is not related to robotic environments. ATTACH concentrates on specific manual motions but does not address task sequences, and it lacks human-robot interference such as occlusions. HA-ViD stresses that the data annotation should be shared between humans and robots, yet it lacks human-robot interference on motion. The dynamic nature of HRS often results in human-robot occlusions in practical scenarios, for example, when a moving robot partially occludes the human body. Such occlusions are seldom present in publicly available datasets of daily human activities or in existing datasets about HRS, limiting the usage of these datasets in practical applications.

On the other hand, human motion processing is particularly pivotal for implementing HRS. From the related work, human motion processing can be categorized into several subfields, including gesture recognition, motion classification, motion prediction, task and procedure prediction, intention recognition, autonomous control of robotic systems, and robotic task procedure generation, among others^16,17^. However, the current limitations of existing work and their experiment conditions are evident in the following aspects:

- **Number of Participants**. Many studies validate proposed methods with few or even a single human due to the scarcity of available participants. For practical HRS applications, the model’s ability to generalize across individuals is crucial.
- **Environmental Condition**. Many precious publications employ relatively simple scenarios where a human interacts with a static environment, without the presence of industrial robots. Many studies only operate under ideal conditions, assuming high-quality data, clear vision, and the absence of occlusions. Human-robot occlusions are often neglected, resulting in methods that perform well only in controlled ideal environments.
- **Hardware Barrier**. Some datasets are constructed using specialized equipment like RGB-D cameras or wearable motion-capturing suits, which are impractical for large-scale deployment of models trained on such datasets in industrial settings.
- **Task Sequences**. In numerous published studies, the categories of human motions are overly simplistic, including actions such as raising hands, crossing arms, and waving hands, which are not typically encountered in real HRC scenarios. Additionally, similar and repetitive motions, along with their corresponding task procedures, receive insufficient attention. Many datasets focus on isolated human motions rather than sequential motions within complete tasks, hindering research on robust recognition across motion category transitions.
- **Data Accessibility**. Most studies in HRS, especially HRC, do not publicly share their datasets, making replication, benchmarking, and implementation challenging for the research community.

These limitations hinder the implementation of HRS in realistic environments and tasks. To this end, our dataset focuses on human motions in sequential assembly and disassembly tasks specially designed for HRC. It highlights samples from multiple individuals in human-robot coexisting environments with occlusions and similarity among task-oriented motion categories.

A comprehensive comparison between the proposed and existing datasets is summarized in Table 1. In detail, this dataset can be used to enhance generalization across diverse participants, incorporate multiple camera perspectives, handle real-world noise and occlusions, utilize accessible hardware setups, and focus on practical, repetitive, and sequential human motions. It includes raw video streams and human skeleton data, along with well-labeled and indexed annotations of various sequential human motions involved in assembly and disassembly tasks. Additionally, it provides complete Python scripts for video clipping, stitching, skeleton generation, data formatting, and labeling, ensuring ease of use and reproducibility. By offering these comprehensive features and being openly accessible, this dataset facilitates replication and benchmarking, thereby advancing the field of HRS, especially HRC. This dataset not only overcomes the limitations of existing datasets by providing robust, real-world scenarios with human-robot occlusions and sequential task procedures but also supports a wide range of research applications, including human motion prediction, robotic task planning, and collaborative system development. In summary, the proposed dataset has the following features.

- It involved 33 individuals of different gender (*F*/*M* = 1/2), clothing, height, and body shape, aged from 22 to 28.
- Two distinct scenarios for both assembly and disassembly tasks were set, each further involving static and dynamic settings depending on whether the robot affects the environment.
- Reflection of uncertainties was considered. All individuals behave according to their personal preferences, and the human body is partially blocked by the moving robot.
- Easy-to-use and easy-to-produce case. In this dataset, we used 3D-printed gear system assembly and disassembly tasks with nine procedures. The gear system (from a previous SIEMENS robot learning challenge) can be easily reproduced by users, and the procedures in this gear system are also feasible to be reassociated or reordered.
- Multiple camera views via contactless perception. Three off-the-shelf webcams were installed at different positions and heights without wearable devices.
- Flexibility. Raw videos with indexed frames, well-clipped human manual procedures, and Python scripts, based on which users can reclip the videos to form the task into different procedural sequences or apply different skeleton and mesh tracking algorithms.
- Multiple purposes. This dataset can support future HRS studies such as action recognition^18^, task sequence prediction, behavioral analysis considering uncertainties, robot task planning, robot motion planning^19^, turn-taking prediction in HRC, partially occluded human pose estimation, multi-source sensor fusion, *etc*.

**Table 1:** A comprehensive comparison between the proposed dataset and the existing related datasets.

Dataset	Comparison of Datasets						
	Task	Industrial Environment	Modalities	Contactless Perception (wearable device free)	Number of Participants	Number of Camera Views	Human-robot Occlusions
MHRI^3^	Human teaching robots with objects	×	Visual and audio	*✓*	10	3	×
HRH^5^	Human-robot hugging	×	Foot pressure, position, orientation	×	33	N/A	×
MHHRI^6^	Personality and engagement in HRI	×	Electrodermal activity and ECG	*✓*	18	4	×
THOR^4^	Human-robot navigation	×	Motion trajectory and eye gaze	×	8	1	×
InHARD^9^	Assembly	*✓*	Visual and skeleton	×	16	3	×
NEMO-Lowlands^7^	Gameful HRI	×	Skeleton	*✓*	428	1	×
HARMONIC^8^	Assistive eating	×	Visual, eye gaze, body pose, hand pose, facial keypoints	×	24	3	×
HRI30^10^	Industrial HRI	*✓*	Visual	*✓*	11	1	×
Assembly101^11^	Assembly and disassembly	*✓*	Hand pose	*✓*	53	8	×
ATTACH^12^	Assembly	*✓*	Visual and skeleton	*✓*	42	3	×
HA-ViD^13^	Assembly	*✓*	Visual	*✓*	30	3	×
**Ours**	Assembly and disassembly	*✓*	Visual and skeleton	*✓*	33	3	*✓*

Furthermore, an extensive evaluation of 13 state-of-the-art deep learning models was conducted across two practical aspects: early human motion prediction (offline) and robot task procedure generation (online). The results indicate a noticeable performance gap between offline and online settings, highlighting the need for further research to enhance online inference with smoother transitions between task procedures. Additionally, the design trade-off between model capacity and computational efficiency expects a more detailed exploration.

## Methods

**Fig. 1:**
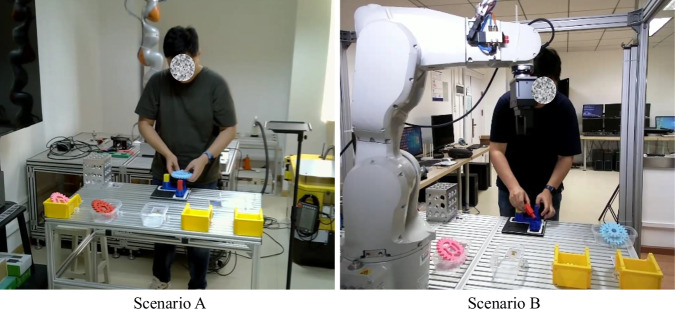
Scenarios for data collection.

The proposed dataset is collected from two scenarios. Scenario A is about fewer human-robot occlusions, while there are more occlusions in scenario B. In both scenarios, containers are placed on a workbench, and product components are in the containers. In scenario B, an industrial robot was moving arbitrarily in front of human operators. The two scenarios are shown in Fig. 1.

**Fig. 2:**
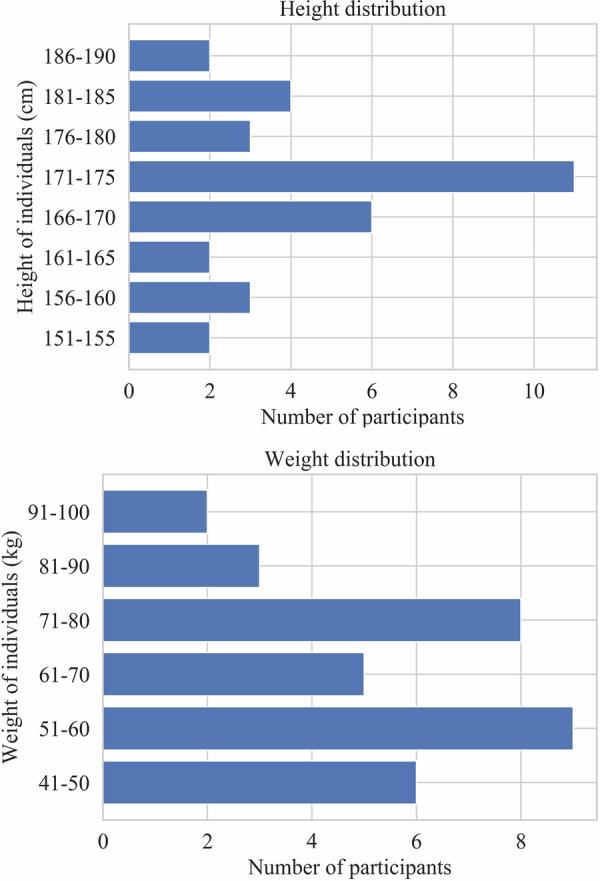
Height and weight distribution of all the participants.

There are 25 participants in each scenario. Because some of the participants only joined a single scenario, the complete dataset contains data from 33 student volunteers aged from 22 to 28. 8 of them are only in scenario A, 8 of them are only in scenario B, and 17 of them are in both scenarios. Among them, 11 are female and 22 are male. The height and weight distribution of all the participants are shown in Fig. 2. All participants were informed about the types of data to be collected, how those data would be stored and processed, and the overall purpose of the study. All participants provided consent for data collection and public release of de-identified videos. Participants had the option to wear face masks during data collection. To ensure privacy, all raw videos in the public dataset have been anonymized by automatically blurring faces. According to the Letter of Compliance (Dnr: HS-2025-2104 KS 4.4.1) provided by the KTH Research Ethics Advisor at the KTH Research Support Office, this research does not require institutional review. Although it involves processing information about living human beings, none of the data is traceable to specific individuals, either directly or indirectly, meaning no personal data is handled. Furthermore, the research does not involve any sensitive personal information such as health data, genetic or biometric data, or information related to ethnicity, beliefs, or legal offences. It also does not involve any physical or psychological procedures on human subjects, nor does it pose any risk of harm or use of biological samples. Therefore, the study falls outside the scope of the Swedish Ethics Review Act and does not require formal ethics approval.

The assembly and disassembly tasks in this dataset use a gear system as the object. It contains seven components corresponding to seven task procedures. In addition, two more procedures are also contained, which are moving the plate from the holder to the workbench and moving the product installed on the plate from the workbench to the endpoint. In all, there are 9 procedures in both tasks. The CAD model of this gear system is also in the dataset as CAD_model.zip, which could be built using 3D printers.

Only off-the-shelf web cameras (Logitech C270 and HIKVISION E14a) were used for data collection. Participants were not required to use any wearable devices. Any video sample in this dataset was recorded by three cameras with different perspectives simultaneously. Cameras were located at different heights arbitrarily. For scenario A, cameras were located at 170 cm (left), 155 cm (middle), and 160 cm (right). For scenario B, cameras were located at 165 cm (left), 155 cm (middle), and 155 cm (right). There is no restriction on lighting conditions or coordinated lighting for multiple cameras.

**Fig. 3:**
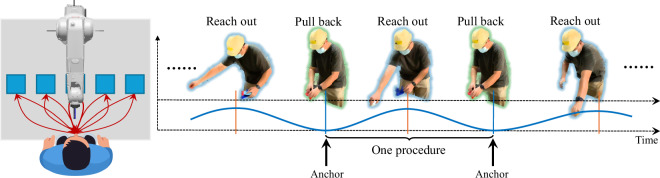
Illustration of the protocol.

During the assembly, the participants are required to first take a plate for components to be installed onto, then take components from containers and conduct assembly task procedures, and finally transport the assembled product with the plate to the endpoint. Disassembly is conducted inversely. Participants are required to conduct one task three times. Before data collection, every volunteer was taught how to do the assembly and disassembly tasks, and given one trial to practice. Apart from what has been mentioned, participants have no other rules to follow. For example, participants can use any hand for any procedure, use one hand or both hands for grasping, and are allowed to hesitate at any time. An illustration of such a protocol is shown in Fig. 3, in which anchors (the frame index dividing two adjacent procedures) are used to divide assembly and disassembly procedures, and the red arrows and blue curve are used to illustrate the reach-out and pull-back motion and trend, respectively. 5 annotators were required to label the start and end frame indexes for every procedure. One of them designed the annotation protocol, led the annotation team, supervised the work of the other four annotators, and finally verified all annotations by reviewing the segmented videos based on the provided frame indices.

## Data Records

The dataset is available at Dryad^20^. This dataset consists of 10,100 task procedure samples in total, that is 5,050 samples for each scenario and 2,025 samples for each task. In each scenario, data are from 25 participants, 3 cameras, 3 times of one task, 2 main tasks (assembly and disassembly), and 9 procedures. A summary of this dataset appears in Table 2. Concretely, this dataset consists of raw videos, clipped videos, and clipped 2D and 3D skeleton data.

**Table 2:** Summary of the dataset.

Scenario	Task	Participants	Processed Samples	Total Frames	Total Time
A	Assembly	25	2,025	224,521	2:15:17
	Disassembly	25	2,025	205,414	1:53:38
B	Assembly	25	2,025	236,576	2:18:34
	Disassembly	25	2,025	212,964	1:58:22
**Total**		**33**	**8,100**	**879,475**	**8:25:51**

### Raw videos

The raw videos in this dataset are with 640 × 480 resolution. All videos are recorded at 30 frames per second, regardless of the camera hardware. All the videos are stored in AVI files, together with TXT files indicating the frame number and time in seconds from the start of the video. Each piece in the TXT files is a tuple containing three elements, that is (*i*, *t*, *T*), in which *i* (index of row) is the index of one frame, *t* is the time from the start in millisecond, and *T* is the clock time when this frame is recorded. There exist 900 raw videos in total, 450 for each scenario, and 225 for each task in one scenario.

### Task procedure anchors

Task procedure anchors are the keyframe numbers that can annotate the start and end of any sequential human motions in the assembly and disassembly tasks. They are stored in CSV files. As for each row in such a CSV file, the first column is the name of one raw video. The remaining are 18 frame anchors (the start and end frames) for 9 task procedures in this raw video. For one task in each scenario, there is one CSV file of the task procedure anchors. The anchors of the annotation were done manually with Adobe Premiere Pro.

### Video clips

Video clips were generated using the raw video and the task procedure anchors by the Python scripts. Every raw video is clipped into 9 procedural video clips. Video clips are also in AVI format and are renamed based on the file name of the raw video. There exist 10,100 video clips in total, 5,050 for each scenario, and 2,025 for each task in one scenario.

### Skeleton frames

Skeleton frames are about the unmerged skeleton data generated by OpenPose (for 2D)^21^ and MMPose (for 3D)^22^ when taking video clips as input. We chose OpenPose for 2D skeleton generation due to its fast processing speed. We selected MMpose for 3D skeleton estimation due to its integration within the OpenMMLab ecosystem and its stronger modeling of human body priors. MMpose leverages parametric human models and applies kinematic constraints to enforce anatomical plausibility, which helps mitigate common issues such as joint floating, bone twisting, or limb penetration. End users of this dataset could switch to any skeleton generator for their usage. For every video clip, there is a series of JSON files representing the skeleton data of each frame. It is also possible to use other skeleton estimators on all the raw videos or video clips. The skeleton frames JSON files are formatted in the following way. This dataset provides skeleton data in both 2D and 3D formats. Within the 2D format, pose_keypoints_2d is heavily used, represented by (*x*, *y*, *c*), where *x*,*y* are the coordinate value, and *c* is the confidence value on this joint by the skeleton estimator. All 18 joints on one frame are listed in pose_keypoints_2d with an order defined by the Microsoft COCO dataset^23^. For the 3D skeleton, only the procedural skeleton sequences are provided. Skeleton frames are aimed at an easier understanding of the skeleton data on each frame, while the procedural skeleton sequences in the following paragraph are more often used.

### Procedural skeleton sequences

A procedural skeleton sequence is a sequence of skeleton data from a single camera within one procedure in a task. One procedural skeleton sequence can be regarded as a sample, with a label indicating the category of this procedure. Procedural skeleton sequences are stored in JSON files. In such 2D skeleton JSON files, data contains frame indexes, as well as pose and score (confidence score generated by OpenPose) data at every frame. Finally, the label and its index are stored at the end of such a JSON file, as well as in the filename. The 3D skeleton JSON files have a similar structure, which can be seen as follows. Normalised procedural skeleton sequences are also provided in this dataset. The normalisation was conducted on camera resolution, for the user to be able to train models running on cameras with different resolutions. The users can certainly flexibly conduct the normalisation here or in the following steps, such as building the NumPy-based files before training.

### Naming rules

All the AVI raw video files and their corresponding TXT files are with such a naming rule that is *SS*_*PP*_*N*_*K*_*C*, in which *SS* ∈ {*s*1, *s*2}, *PP* ∈ {*p*1, *p*1, …, *p*25},*N* ∈ {1, 2, 3}, *K* ∈ {*a*, *d*}, and *C* ∈ {1, 2, 3} are labels for scenarios, participant number, trial number, task, and camera number, respectively. Concretely, *s*1 is scenario A and *s*2 is scenario B. As for the task, *a* represents the assembly task while *d* is the disassembly task. Regarding cameras, camera 1 is the one to the right front of the human, camera 2 is the one to the left front of the human, and camera 3 is the one in the centre. All the AVI video clips were renamed by adding a final symbol to the original raw video name. It is shown as *SS*_*PP*_*N*_*K*_*C*_*RR*, where *RR* ∈ {*c*1, *c*2, …, *c*9}. Skeleton frames are named by *SS*_*PP*_*N*_*K*_*C*_*RR*_*F*, where *F* is the frame index starting from 0. Procedural skeleton sequences merged by skeleton frames are also named by *SS*_*PP*_*N*_*K*_*C*_*RR*.

## Technical Validation

Extensive validations have been performed based on this dataset to demonstrate the practical usage of this dataset quantitatively and establish the benchmark for future research works. Concretely, a variety of state-of-the-art action recognition models in the computer vision field are implemented to realize the early prediction of human sequential motions for further task procedure generation for robots, inspired by previous work^24^ that used only the 2D skeleton data in the assembly task. These models include the RGB video-based ones (C3D^25^, I3D^26^, SlowFast^27^, TPN^28^, TimeSformer^29^, VideoSwin^30^, MViT^31^) and the skeleton-based ones (STGCN^32^, 2s-AGCN^33^, PoseC3D^34^, STGCNPP^35^). These models represent the major developments of human action recognition, *e.g*., from earlier Convolutional Neural Network (CNN)-based ones such as C3D and I3D to recent Transformer-based ones such as VideoSwin and MViT. They have also shown promising performances on various benchmarks in the general domain. For the skeleton-based models, Graph Convolutional Network (GCN) is commonly considered because it well captures the topological patterns of human body skeleton. Both 2D skeleton data generated from OpenPose and 3D skeleton data generated from MMPose are considered as the input modality. We benchmark both video and skeleton-based methods because of the consideration of the communication bandwidth cost of robotic applications. Often, skeleton-based modality has much lower data volume and requires fewer computational resources. The computer used for validation was equipped with an Intel i9-13900KF CPU, an RTX4090 GPU, and 64GB of RAM.

### Human Motion Early Prediction

Human motion early prediction aims to recognize the task-related category of each human motion before it is completed, *i.e*., using a portion of video frames as the available observations. The fundamental problem is to classify the video segments based on the RGB or skeleton pose information.

The algorithmic performance for the early prediction of sequential human motions is evaluated for each of these models. Four subsets of this dataset are individually employed to report the outcomes (assembly and disassembly of scenario A and scenario B). Instead of randomly splitting each subset into the training and testing parts, two practical schemes are adopted that are similar to those in NTU RGB+D^15^: X Subject and X View. For X Subject, 70% of the entire participants are considered in training while the remaining 30% are used for testing. For X View, cameras 2 and 3 are considered in training while camera 1 is used for testing. Such schemes aim to fully exploit the diversity presented in this dataset, thus bridging the gap between the existing datasets and the HRC cases in the real world. Moreover, the first 25% and 50% of each of the clipped entire human motions are provided as the available information for the models, respectively. This is challenging yet meaningful because the robot task procedures need to be generated as early as possible to ensure smooth procedural transitions between human and robot. Top-1 and Top-2 recognition accuracies are reported for each of the configurations. The number of floating operations (FLOPs) of each implemented model is also presented (in the unit of Giga) to reflect the computational efficiency.

Tables 3–6, below show the results on the four subsets of this dataset, respectively. In general, it can be noticed that scenario B is more challenging than scenario A, regardless of the assembly or disassembly.

**Table 3:** Human motion prediction results for all implemented models (Scenario B, assembly).

Model	X Subject				X View				FLOPs
	25%		50%		25%		50%		
	Top1	Top2	Top1	Top2	Top1	Top2	Top1	Top2	
C3D	98.77	99.54	98.92	99.54	49.19	67.11	52.30	69.63	38.50
I3D	98.61	99.85	99.23	100.00	77.19	83.85	66.81	85.63	43.50
SlowFast	91.20	96.60	97.07	99.07	57.78	84.74	68.89	82.81	65.71
TPN	98.75	100.00	99.23	100.00	73.04	93.63	73.78	96.30	66.01
TimeSformer	96.45	99.54	96.14	99.38	81.48	95.11	79.26	94.52	141.00
VideoSwin	**98.92**	**99.85**	**99.38**	99.79	**86.22**	**95.89**	**88.59**	**96.61**	166.00
MViT	95.99	97.99	99.07	**100.00**	75.11	89.63	86.07	93.04	64.00
STGCN-2D	58.49	75.46	**93.52**	**97.68**	13.63	34.37	21.78	32.15	3.80
2s-AGCN-2D	78.70	88.58	90.90	96.91	29.93	39.70	29.78	46.52	4.40
PoseC3D-2D	76.54	89.20	81.94	93.06	29.33	42.81	33.93	50.07	20.60
STGCNPP-2D	**83.80**	**91.82**	90.61	97.02	16.74	27.85	34.22	48.74	1.95
STGCN-3D	72.38	83.95	81.64	93.21	71.45	82.72	79.63	90.76	5.70
STGCNPP-3D	76.08	88.58	86.42	96.30	**77.78**	**88.27**	**86.42**	**95.83**	2.96

**Table 4:** Human motion prediction results for all implemented models (Scenario B, disassembly).

Model	X Subject				X View				FLOPs
	25%		50%		25%		50%		
	Top1	Top2	Top1	Top2	Top1	Top2	Top1	Top2	
C3D	95.22	97.69	99.07	99.85	61.48	80.74	54.37	77.04	38.50
I3D	97.38	99.38	97.06	99.23	63.85	85.33	84.74	93.63	43.50
SlowFast	96.76	98.92	96.91	98.15	85.19	91.56	89.93	96.44	65.71
TPN	97.84	99.38	97.69	99.04	87.85	95.85	89.78	97.19	66.01
TimeSformer	95.99	98.92	97.99	99.63	87.70	96.52	78.22	91.77	141.00
VideoSwin	97.69	99.81	**99.38**	**100.00**	91.08	96.43	90.52	**98.64**	166.00
MViT	**98.61**	**99.85**	98.77	99.89	**93.34**	**98.06**	**92.71**	98.57	64.00
STGCN-2D	65.90	84.88	83.64	93.36	28.19	42.37	33.48	42.65	3.80
2s-AGCN-2D	65.12	81.79	84.26	**95.99**	38.37	56.59	31.85	40.89	4.40
PoseC3D-2D	70.83	84.10	83.49	93.37	38.04	53.93	40.63	56.15	20.60
STGCNPP-2D	70.68	85.19	**86.57**	95.83	39.96	57.19	39.85	53.41	1.95
STGCN-3D	65.12	82.10	76.23	87.65	65.90	80.56	79.17	92.13	5.70
STGCNPP-3D	**71.14**	**85.49**	80.56	91.67	**70.68**	**85.34**	**82.72**	**92.75**	2.96

**Table 5:** Human motion prediction results for all implemented models (Scenario A, assembly).

Model	X Subject				X View				FLOPs
	25%		50%		25%		50%		
	Top1	Top2	Top1	Top2	Top1	Top2	Top1	Top2	
C3D	97.69	99.38	99.07	99.69	90.96	98.52	98.37	99.70	38.50
I3D	98.30	99.54	**99.38**	99.71	99.56	100.00	98.34	99.58	43.50
SlowFast	97.07	98.61	97.53	98.30	98.81	99.85	97.04	99.26	65.71
TPN	**99.08**	**99.54**	98.92	99.85	99.41	100.00	**100.00**	**100.00**	66.01
TimeSformer	96.14	98.77	97.53	99.54	96.30	99.70	99.11	100.00	141.00
VideoSwin	98.30	99.53	98.92	**99.86**	**100.00**	**100.00**	99.26	100.00	166.00
MViT	97.69	99.23	98.61	99.38	99.85	100.00	99.87	100.00	64.00
STGCN-2D	77.01	88.12	81.48	90.90	68.44	80.89	64.30	79.26	3.80
2s-AGCN-2D	87.96	94.60	90.28	96.60	65.63	80.04	63.41	80.15	4.40
PoseC3D-2D	85.19	93.21	81.33	92.59	70.22	87.11	66.37	88.19	20.60
STGCNPP-2D	89.35	94.44	88.89	96.30	56.30	73.62	77.33	90.37	1.95
STGCN-3D	88.73	94.60	90.74	96.14	84.26	91.82	92.90	**97.53**	5.70
STGCNPP-3D	**90.75**	**96.91**	**96.13**	**97.86**	**92.90**	**96.14**	**94.29**	96.91	2.96

**Table 6:** Human motion prediction results for all implemented models (Scenario A, disassembly).

Model	X Subject				X View				FLOPs
	25%		50%		25%		50%		
	Top1	Top2	Top1	Top2	Top1	Top2	Top1	Top2	
C3D	98.46	100.00	99.54	100.00	95.85	99.70	99.56	100.00	38.50
I3D	99.54	100.00	99.86	100.00	99.11	99.41	95.17	99.13	43.50
SlowFast	97.84	99.52	100.00	100.00	98.07	99.70	99.56	100.00	65.71
TPN	99.85	100.00	100.00	100.00	99.71	100.00	**100.00**	**100.00**	66.01
TimeSformer	97.06	99.69	97.22	99.85	97.63	100.00	97.66	99.89	141.00
VideoSwin	**100.00**	**100.00**	**100.00**	**100.00**	99.41	100.00	100.00	100.00	166.00
MViT	99.54	100.00	99.69	99.85	**100.00**	**100.00**	99.70	100.00	64.00
STGCN-2D	70.52	85.34	83.64	94.29	42.37	61.84	55.86	65.19	3.80
2s-AGCN-2D	72.38	87.19	**92.13**	**97.53**	51.41	69.78	67.56	80.74	4.40
PoseC3D-2D	65.43	78.55	82.87	91.98	68.30	81.04	65.48	85.93	20.60
STGCNPP-2D	**77.01**	87.04	89.51	95.99	59.85	81.19	70.96	89.48	1.95
STGCN-3D	73.30	84.26	84.41	93.67	65.12	78.40	85.49	**94.75**	5.70
STGCNPP-3D	74.23	**88.89**	84.26	93.83	**72.53**	**86.42**	**87.96**	94.60	2.96

### Robot Task Procedure Generation

Robot task procedure generation indicates generating a procedure in a task sequence for the robot to conduct collaboratively, given the current status of the human motion. It stresses the online inference performance of each implemented model, which is directly related to the efficiency of HRC. Specifically, the models here are requested to execute the real-time prediction (inference) once every 5 video frames with a temporal sliding window of 32 video frames on each complete task video (instead of the procedural videos in the previous validation). The task sequence constraints (the gear system mentioned before) for the robot task procedure generation validation are from previous work^24^.

For each of these models, two important performance-related values are reported for each atomic human task procedure plus the average overall task procedures from Tables 7–10. The first value (the upper rows) corresponds to the temporally-aggregated prediction accuracy, while the second value (the lower rows) corresponds to the degrees of delay for the first correct prediction. Concretely, the first value quantifies the percentage of correct prediction times with respect to the overall attempts made. It is different from algorithmic performance in that it concerns continuous predictions as well as the overlap between successive procedures. The second value quantifies the percentage of running video frames before a correct prediction result first appears. It is intended to reflect how timely a meaningful robot task procedure can be generated based on human motion prediction. Tables 7–10 below present the results of the online inference performance for the challenging X View scheme and 50% observation of the complete videos. Considering the page limit, only the RGB video-based models are involved here because they generally perform better than the skeleton-based models.

**Table 7:** The online inference results related to robotic procedure generation (Scenario B, assembly, X View, 50% Observation).

Model	P0	P1	P2	P3	P4	P5	P6	P7	P8	All
Temporally-aggregated prediction accuracy ↑										
C3D	8.37	73.60	74.27	19.97	0.24	37.34	77.85	8.78	96.25	44.08
I3D	38.72	10.09	71.46	76.40	37.04	66.07	75.78	66.55	77.27	57.71
SlowFast	53.47	69.15	35.13	13.22	74.99	2.98	79.27	9.82	68.29	45.15
TPN	99.75	41.22	34.35	91.87	46.31	12.71	81.76	51.25	60.70	57.77
TimeSformer	66.97	66.13	25.95	87.12	34.27	72.49	69.90	26.61	51.14	55.62
VideoSwin	98.79	64.48	80.33	70.42	66.59	61.68	97.75	21.25	82.35	**71.52**
MViT	45.80	84.00	48.23	16.90	71.84	64.45	24.63	35.59	32.66	47.12
**Degrees of delay for first correct prediction↓**										
C3D	19.70	8.94	5.21	37.05	70.03	11.31	1.37	51.17	2.46	23.03
I3D	31.58	69.57	20.71	22.49	41.34	18.53	6.15	27.02	23.83	29.02
SlowFast	25.43	14.67	28.20	57.25	15.71	46.00	5.14	38.93	15.51	27.43
TPN	4.92	25.99	4.80	1.51	37.06	37.46	1.23	7.82	22.04	15.87
TimeSformer	5.49	5.03	10.34	2.91	25.89	6.27	0.79	5.68	0.00	**6.93**
VideoSwin	21.78	30.28	14.20	26.10	26.18	27.19	2.45	61.31	1.60	23.45
MViT	16.85	3.39	16.79	33.77	6.41	16.74	25.83	27.62	16.00	18.16

**Table 8:** The online inference results related to robotic procedure generation (Scenario B, disassembly, X View, 50% Observation).

Model	P0	P1	P2	P3	P4	P5	P6	P7	P8	All
Temporally-aggregated prediction accuracy ↑										
C3D	77.20	75.64	68.31	18.74	9.72	44.50	42.56	33.02	21.17	43.43
I3D	83.77	49.13	93.97	53.41	51.03	57.95	48.18	35.31	87.86	62.29
SlowFast	82.55	82.56	59.51	89.58	74.65	23.99	4.15	48.47	88.93	61.60
TPN	81.85	67.10	28.14	46.77	89.97	11.99	51.58	6.60	50.66	48.30
TimeSformer	73.53	67.19	55.60	75.50	34.45	67.01	20.10	64.26	87.92	60.62
VideoSwin	72.75	99.42	67.52	61.13	27.85	94.58	59.36	69.48	90.65	**71.41**
MViT	50.54	70.89	45.65	77.76	50.41	30.45	55.39	70.22	61.34	56.96
**Degrees of delay for first correct prediction ↓**										
C3D	7.71	3.44	11.26	32.66	16.26	29.74	22.99	22.43	32.62	19.90
I3D	16.17	11.63	0.00	39.56	40.69	38.86	47.43	45.32	13.37	28.12
SlowFast	15.94	8.10	13.78	5.01	15.65	61.05	34.30	42.94	7.54	22.70
TPN	3.61	3.00	9.89	5.16	3.44	26.11	10.44	26.33	1.22	9.91
TimeSformer	3.74	1.86	2.99	4.55	9.99	4.02	19.85	15.51	0.41	**6.99**
VideoSwin	16.17	0.57	22.55	37.97	42.76	5.13	32.52	29.75	11.55	22.11
MViT	14.73	0.87	6.57	9.00	23.68	34.30	17.14	11.68	12.37	14.48

**Table 9:** The online inference results related to robotic procedure generation (Scenario A, assembly, X View, 50% Observation).

Model	P0	P1	P2	P3	P4	P5	P6	P7	P8	All
Temporally-aggregated prediction accuracy ↑										
C3D	76.72	66.66	87.39	72.29	90.84	83.06	84.15	83.29	86.55	81.22
I3D	95.07	61.27	92.08	92.38	77.07	46.57	92.07	65.67	67.17	76.59
SlowFast	91.36	53.57	60.82	76.15	81.13	60.81	73.20	76.83	47.37	69.03
TPN	41.02	75.50	75.12	70.39	99.44	28.22	98.02	68.92	77.66	70.48
TimeSformer	69.78	71.51	72.73	68.41	89.81	61.17	96.28	55.16	94.65	75.50
VideoSwin	86.82	96.70	79.27	77.21	79.43	84.60	82.13	84.12	84.68	**83.88**
MViT	48.28	92.03	83.36	53.44	94.47	60.39	97.21	77.14	79.79	76.23
**Degrees of delay for first correct prediction ↓**										
C3D	9.50	13.52	0.99	9.88	4.05	11.10	12.10	21.81	12.38	10.59
I3D	19.98	18.12	1.41	4.71	24.54	37.20	8.19	56.64	36.56	23.04
SlowFast	20.39	42.52	28.18	14.44	19.60	38.00	23.51	36.77	34.08	28.61
TPN	13.45	2.28	0.32	1.93	0.48	23.72	0.51	32.41	19.22	10.48
TimeSformer	4.41	2.28	0.08	5.75	3.04	7.12	1.05	19.29	1.41	**4.94**
VideoSwin	19.91	0.67	21.40	20.25	17.54	10.06	11.81	21.87	16.60	15.57
MViT	24.24	3.04	7.78	22.30	2.42	22.85	1.91	32.33	0.00	12.23

**Table 10:** The online inference results related to robotic procedure generation (Scenario A, disassembly, X View, 50% Observation).

Model	P0	P1	P2	P3	P4	P5	P6	P7	P8	All
Temporally-aggregated prediction accuracy ↑										
C3D	73.96	96.03	83.87	86.48	87.08	92.22	61.15	65.80	96.58	**82.58**
I3D	93.29	96.73	76.47	90.03	68.82	38.86	16.07	60.23	62.18	66.97
SlowFast	86.59	96.28	61.07	94.62	51.52	48.11	24.90	43.98	63.77	63.42
TPN	72.93	62.94	78.17	73.16	53.00	99.07	58.78	41.43	62.09	66.84
TimeSformer	70.57	61.07	72.87	79.35	95.58	83.59	70.34	76.03	93.84	78.14
VideoSwin	86.28	98.45	87.68	87.79	69.89	80.29	44.99	71.96	85.84	79.24
MViT	80.74	96.57	94.57	74.98	78.61	72.24	35.45	64.55	78.28	75.11
**Degrees of delay for first correct prediction ↓**										
C3D	7.91	0.07	13.03	6.33	5.90	5.39	33.23	22.61	2.31	10.75
I3D	16.34	2.43	11.97	4.61	29.78	61.50	74.45	39.28	39.96	31.15
SlowFast	16.38	3.70	29.15	4.60	44.46	51.27	72.86	50.81	33.34	34.06
TPN	4.06	0.52	0.79	1.69	6.32	0.00	28.32	23.38	20.65	9.53
TimeSformer	3.69	0.00	4.93	1.33	1.90	13.09	21.58	17.56	1.96	**7.34**
VideoSwin	16.34	0.86	11.57	11.48	29.62	19.97	54.80	27.74	16.01	20.93
MViT	8.61	0.59	2.94	16.83	14.13	17.00	35.88	17.59	17.63	14.58

## Usage Notes

Users can use the data from different folders for their research purposes. For instance, for each task in one scenario, users can reach raw video and its corresponding frame index and timestamp from folders raw video and raw video frames. Data from these two folders can be used for video processing and further data generation with the specific needs of the users, just like skeletons. Data in folders procedural skeleton sequences and procedural skeleton sequences 3D are about the human skeleton sequences of every task procedure, which can be used for skeleton-based processing. Human skeletons were generated by estimators with no bias or further manual tuning. It means all the raw videos are the ground truth, while the quality of human skeletons relies on the human skeleton estimator. Occlusions affect the precision of skeleton joint position and may cause loss of joints. Users could switch to any other skeleton estimators for their research. Data in procedure_anchors.csv can be used to clip the raw video into procedural ones. Data from the aforementioned two scenarios can also be used jointly, for instance, to study the effects on parameter sensitivity by environmental changes. Because this dataset provides divided procedural data, rearranging the sequence can boost the diversity regarding the procedure sequence.

## Data Availability

The dataset has been deposited to Dryad: 10.5061/dryad.ncjsxkt6f.

## Code availability

The following GitHub repository contains the Python scripts to operate and use this dataset: https://github.com/KTH-IPS/SD-Dataset.
